# Tea Polyphenols Relieve the Fluoride-Induced Oxidative Stress in the Intestinal Porcine Epithelial Cell Model

**DOI:** 10.3390/toxics13020083

**Published:** 2025-01-24

**Authors:** Chunyan Xie, Shuyi Niu, Wen Tian

**Affiliations:** 1 Tianjin Key Laboratory of Animal Molecular Breeding and Biotechnology, Tianjin Livestock and Poultry Health Breeding Technology Engineering Center, Institute of Animal Science and Veterinary, Tianjin Academy of Agricultural Sciences, Tianjin 300381, China; xie.chunyan@foxmail.com; 2 Key Laboratory of Agro-ecological Processes in Subtropical Region, Institute of Subtropical Agriculture, Chinese Academy of Sciences, Changsha 410125, China; niu461130365@foxmail.com

**Keywords:** fluorosis, TPs, oxidative stress, ROS, IPEC-J2 cells

## Abstract

Prolonged excessive intake of fluoride (F) can result in fluorosis, leading to a range of tissue oxidative damages. Therefore, mitigating the oxidative stress induced by fluorosis has become a significant research concern. Consequently, how to relieve oxidative stress caused by fluorosis is an urgent matter. In the present study, intestinal porcine epithelial (IPEC-J2) cells were chosen to explore the underlying mechanism of tea polyphenols (TPs) on F-induced oxidative stress. The results show that the cytotoxicity of IPEC-J2 cells induced by F presented a dose-dependent manner according to cell viability. Additionally, F treatment inhibited the activity of T-SOD, CAT, and GSH-Px as well as their transcription levels, increased the reactive oxygen (ROS) formation and cell damage rates, and then promoted cell apoptosis through the results of TUNEL and mitochondrial membrane potential detection when compared with the IPEC-J2 cells from the control group. As the main antioxidant ingredient in tea, TPs alleviated F-induced cell oxidation and apoptosis via blocking F-induced ROS generation and LDH’s release, as well as promoting the transcription of tight junction (TJ) proteins and the activities of antioxidant enzymes in IPEC-J2 cells. These results provide a new treatment strategy for F-induced intestinal oxidative impairment.

## 1. Introduction

Humans and animals can be exposed to F through drinking water, air, and food supplements. However, long-term excessive F intake would cause fluorosis, which might disrupt the redox equilibrium, produce more free radicals, and then cause significant oxidative damage in bones, teeth, the gastrointestinal tract, etc. [1,2]. Many studies showed that excessive F impairs antioxidant defense systems [3,4]. F accumulation could increase ROS formation [5,6] and reduce antioxidant enzyme activity [3,6]. It has been reported that NaF treatment significantly increases the hepatic MDA level in mice and induces the generation of free radicals, thus leading to intracellular mitochondrial damage [7,8]. F overexposure in the diet could reduce the serum antioxidant capacity, destroy the intestinal barrier, and increase the intestinal permeability of mice [9]. However, there are still no oxidation-free and effective treatment methods for fluorosis, which has become a global public health problem in several nations, including China [10].

It is well known that antioxidants could be an effective strategy for maintaining intestinal health [11]. As one of the most popular drinks worldwide, tea is rich in polyphenols, which are the major active compounds present in teas [12]. It has been proven many times that TPs have anti-oxidant activity and stress resistance properties thanks to their ability to limit ROS formation by inhibiting the activity of oxidative enzymes, improve the activity of endogenic antioxidants, and sweep ROS, indicating they play a key role in regulating free radicals [13,14]. TPs could activate different antioxidant enzymes and attenuate the oxidative damage of DNA. Apart from this, TPs are also regarded as a potent stress regulator in mediating the stress induced by metal ions via ROS scavenging [11,15]. It has been reported that TPs in green tea could prevent some metal-induced oxidative stress and damage [16]. However, few studies are available linking TPs to the occurrence of fluorosis, and we believe that this issue should be looked into, given the proven oxidation resistance-promoting effect of TPs.

Generally, F is transported to various tissues of the body after being absorbed by the gastrointestinal tract. As the main organ exposed to nutrients and toxic food contaminants, the defenses of the intestine are not enough to respond adequately against the oxidative stress and contribute to developing intestinal pathologies under conditions which exacerbate ROS production [17,18]. Moreover, aside from residents, pigs breeding in most fluoride-rich areas still have to face the F threat from local underground water. Therefore, based on the fact that IPEC-J2 cells are widely used to study the impacts of nutrients or toxins on intestinal epithelial cell permeability and intestinal function [19,20], we aimed to explore the underlying mechanism by which TPs eliminate intestinal oxidative stress induced by F exposure in IPEC-J2 cells in this study.

## 2. Materials and Methods

This study was conducted in accordance with the Basic & Clinical Pharmacology & Toxicology policy for experimental and clinical studies (2023) [21].

### 2.1. Cells and Reagents

IPEC-J2 cells (BeNa Culture Collection, Beijing, China) were cultured in DMEM + 10% FBS + 100 U/mL penicillin and 0.1 mg/mL streptomycin (Gibico, New York, NY, USA) at 37 °C with 5% CO_2_ throughout the experiment. A cell culture was performed using 12 well Costar Snapwell inserts (Corning Inc., Corning, NY, USA) from 2.5 × 10^5^ to 4.0 × 10^5^ per well in a 0.5 mL volume. The IPEC-J2 cells were allowed to grow for about 36 h to reach an average cell density of 2.5 × 10^5^ per well. Then, the subsequent analysis was conducted.

TPs (purity ≥ 98%) were purchased from Jiangshu Ruixiang Biotechnology Co., Ltd. (Changzhou, China), and NaF (purity ≥ 98%) was purchased from Maclin Biochemical Technology Co., Ltd. (Shanghai, China).

### 2.2. Establishment of NaF-Treated Cell Model and Determination of Working Concentration of TPs

IPEC-J2 cells were cultured in 96 well Costar Snapwell inserts (Corning Inc., Corning, NY, USA), and the remaining procedures followed the same protocol described in Section 2.1. Based on previous studies, we selected the approximate concentrations of the NaF (0 mM, 1 mM, 2 mM, 3 mM, and 4 mM) and TPs (0, 100, 200, 300, and 400 mg·L^−1^) to establish the F-induced IPEC-J2 cell model protected by TPs [22,23,24]. After the IPEC-J2 cells were incubated with a series of NaF for 24 h, we determined the working concentration of NaF according to the cell viability (about 50%), which was found by testing with Cell Counting Kit-8 (CCK8, Dojindo Molecular Technologies, Inc., Kumamoto, Japan). The TP working solution was conformed via the cell viability of F-induced cells.

### 2.3. Cell Treatment

According to studies about the well-known role of TPs in maintaining intestinal health as antioxidants [11,12,13,14,15,18,20], we just divided the IPEC-J2 cells into three groups without the TP group alone—a control group (Control, cultured with medium without FBS for 24 h), NaF group (NaF, treated with 4 mM NaF for 24 h), and NaF +TPs group (NaF +TPs, treated with 4 mM NaF and 200 mg·L^−1^ TPs for 24 h)—after the IPEC-J2 cells were cultured in 12 well Costar Snapwell inserts until the cell confluency reached 80%. Next, we collected cell samples and stored them at −80 °C for the following analysis.

### 2.4. Determination of Antioxidant-Related Parameters

The activities of the total superoxide dismutase (T-SOD), catalase (CAT), and glutathione peroxidase (GSH-Px) were measured with SOD activity assay kit, CAT activity assay kit and GPX activity assay kit from Beijing Boxbio Science & Technology Co.,Ltd. (Beijing, China). After incubation as described in Section 2.3, the cells were harvested into the extract solution of the corresponding kit after being washed with ice-cold and sterile PBS. Then, the supernatant was collected after being centrifuged at 8000× *g* at 4 °C for 10 min for subsequent analysis according to the instructions of the kits. The absorbance was measured with a microplate reader (Infinite M200 PRO, Tecan Trading AG, Shanghai, China).

### 2.5. Intracellular ROS, Cell Apoptosis, and Mitochondrial Membrane Potential (MMP) Detection

The intracellular ROS, cell apoptosis, and MMP of the IPEC-J2 cells were detected using an ROS assay kit, a one-step TUNEL apoptosis assay kit, and an MMP assay kit with JC-1 (Beyotime Biotechnology, Shanghai, China) after being collected as described in Section 2.4. Then, they were detected in accordance with their introductions. The fluorescence intensity is analyzed by Image J software (version 1.53a, National Institutes of Health, Bethesda, MD, USA).

### 2.6. Lactate Dehydrogenase (LDH) Detection

The relative cell damage rate was evaluated via the LDH in the cytoplasm releasing into the medium when the cell membrane was damaged. The LDH level in the medium was detected in accordance with the instructions of the Cytotoxicity LDH Assay Kit-WST^®^ (Dojindo Molecular Technologies, Inc., Kumamoto, Japan) to evaluate the cell membrane permeability via determining the relative cell damage rate.

### 2.7. Quantitative Real-Time PCR (RT-qPCR)

The total RNA of the IPEC-J2 cells was extracted using Trizol reagent (Tiangen Biotech Co., Ltd., Beijing, China), and reverse transcribed with a prime script RT reagent kit (Takara Biotechnology Co., Ltd., Dalian, China). The primer sequences for GPx-1, GPx-2, GPx-3, GPx-4, CAT, SOD, Occludin, Claudin-1, ZO-1, and GAPDH are listed in Table 1. The relative mRNA levels of the genes were calculated with the 2^−ΔΔCt^ method in the case of GAPDH as an internal control.

### 2.8. Statistical Analysis

Data were analyzed by one-way ANOVA in SPSS statistics 21 (SPSS Institute, Inc., New York, NY, USA) and presented as the mean ± SEM. LSD was used for multiple comparisons, where *p* < 0.05 was considered to have a statistical difference and *, **, and *** represent *p* < 0.05, *p* < 0.01, and *p* < 0.001, respectively. GraphPad prism 8.3 (GraphPad Software, San Diego, CA, USA) was utilized to create bar charts and cell survival curves.

## 3. Results

### 3.1. TPs Increased the Viability of IPEC-J2 Cells Induced by F

**Figure 1:**
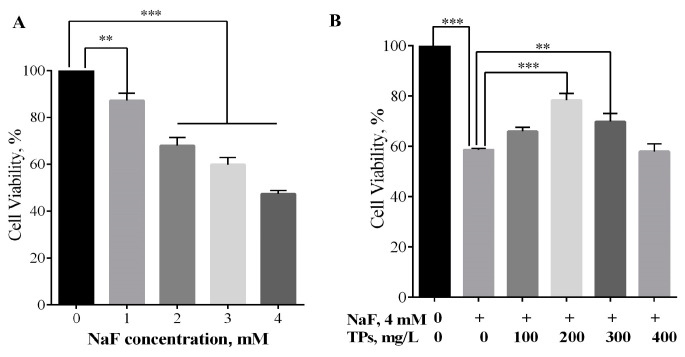
The viability of IPEC-J2 cells treated with different concentrations of NaF or TPs. (**A**) Effect of different concentrations of NaF on the viability of IPEC-J2 cells (*n* = 3). (**B**) Protect effect of different concentrations of TPs on the viability of IPEC-J2 cells (*n* = 3). Data were presented as mean ± SEM. ** *p* < 0.01. *** *p* < 0.001.

The IPEC-J2 cells were exposed to NaF at different concentrations (0 mM, 1 mM, 2 mM, 3 mM, and 4 mM). As shown in Figure 1A, 4 mM NaF was chosen as the inducing solution when the viability of the cells dropped to about 50% (*p* < 0.001). Then, we found that 200 mg·L^−1^ of TPs had the best effect on increasing the viability of F-induced cells (*p* < 0.001) (Figure 1B). Therefore, we used 4 mM NaF and 200 mg·L^−1^ of TPs as the working concentrations in the next series of cell experiments.

### 3.2. Effect of TPs on REDOX-Related Indexes in F-Induced IPEC-J2 Cells

**Figure 2:**
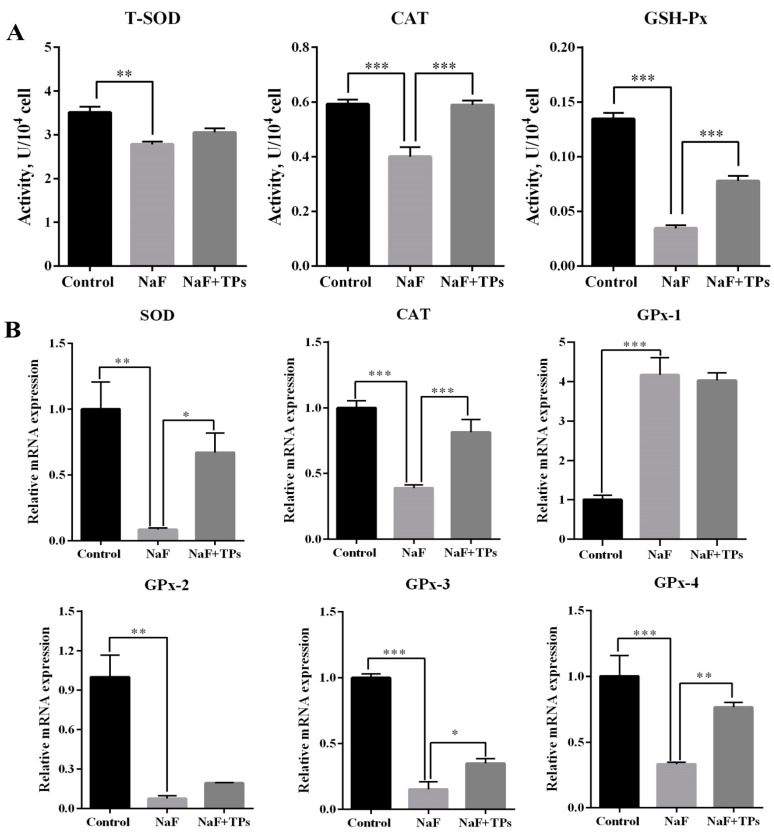
Determination of REDOX-related indexes in IPEC-J2 cells. (**A**) Activity of antioxidant related enzymes (*n* = 3). (**B**) Relative mRNA expression of genes associated with antioxidant enzymes in IPEC-J2 cells (*n* = 3). * *p* < 0.05. ** *p* < 0.01. *** *p* < 0.001.

As shown in Figure 2A, NaF induction inhibited (*p* < 0.01) the activity of CAT, T-SOD, and GSH-Px in cells when compared with the control group, but TP supplementation improved (*p* < 0.001) the activity of CAT and GSH-Px in the F-induced IPEC-J2 cells. The RT-qPCR results also showed a similar trend in the mRNA levels of CAT, SOD, GPx-2, GPx-3, and GPx-4. However, GPx-1 expression was significantly increased (*p* < 0.001) by the NaF treatment and not altered by TP supplementation in the NaF-induced IPEC-J2 cells (Figure 2B), indicating that TPs did not relieve F-induced oxidative stress in the IPEC-J2 cells via regulating the level of GPx-1.

**Figure 3:**
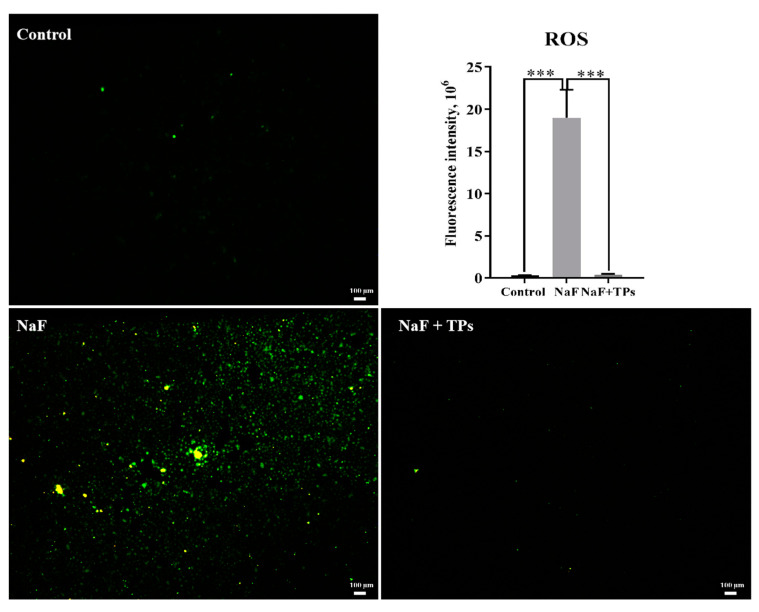
ROS levels in IPEC-J2 cells (*n* = 3). Data were presented as mean ± SEM. *** *p* < 0.001.

The ROS in the IPEC-J2 cells were detected by immunofluorescence after treatment with TPs and NaF for 24 h, which was highly relevant to the level of antioxidant enzymes. The ROS level significantly increased after NaF induction (*p* < 0.001) when compared with the control group but almost reduced to a normal level under TP supplementation of the NaF-induced cells (Figure 3).

### 3.3. Effects of TPs on the Cell Injury-Related Indexes in F-Induced IPEC-J2 Cells

**Figure 4:**
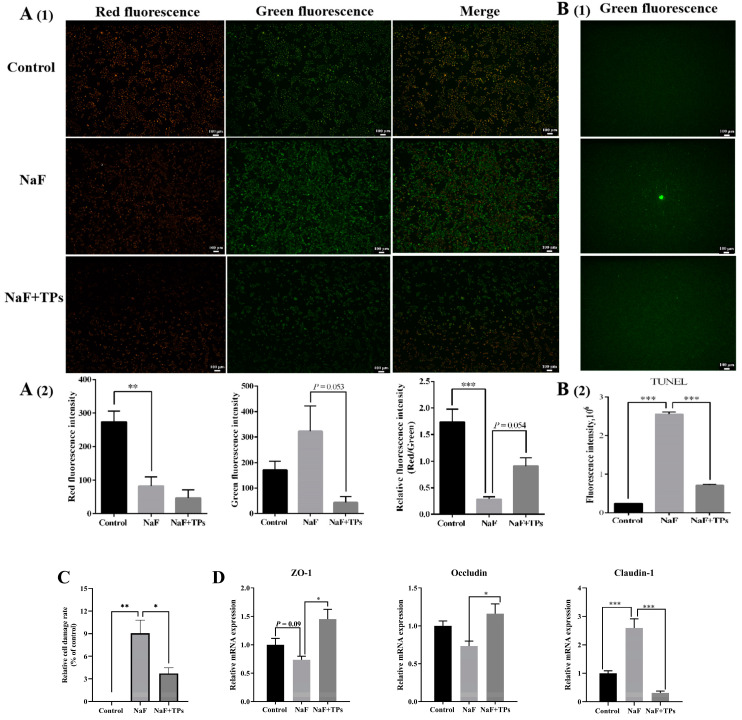
The cell injury-related indexes in IPEC-J2 cells. (**A**) Mitochondrial membrane potential detection with JC-1 in IPEC-J2 cells (*n* = 4): (**1**) Red fluorescence and green fluorescence reflect the high and low MMP of mitochondria, respectively; (**2**) The relative ratio of red fluorescence to green fluorescence in IPEC-J2 cells, and the decrease in the red/green fluorescence ratio indicates that mitochondria are damaged or the cells are in the early apoptosis state. (**B**) TUNEL detection (*n* = 3): (**1**) Fluorescent imagines show apoptotic IPEC-J2 cells, (**2**) Fluorescence intensity analysis in IPEC-J2 cells (**C**) The relative IPEC-J2 cell damage rate according to the LDH level in the medium (*n* = 3). (**D**) The relative mRNA levels of TJ proteins in IPEC-J2 cells (*n* = 3). Data presented as mean ± SEM. * *p* < 0.05. ** *p* < 0.01. *** *p* < 0.001.

Both TUNEL and MMP detection are key indicators in evaluating cell apoptosis. According to the introduction of the TUNEL kit, apoptosis was determined by the relative fluorescence intensity (red/green). As shown in Figure 4A (1,2), NaF induction alone significantly reduced the red fluorescence intensity (*p* < 0.01) and increased the green fluorescence intensity (*p* < 0.001), which resulted in an obvious decrease in the relative fluorescence intensity (red/green) (*p* < 0.001). As expected, TP supplementation notably relieved the downward trend in the relative fluorescence intensity (red/green) (*p* < 0.01) when compared with that in the NaF group. Aside from MMP detection, TUNEL also showed similar results in apoptosis. As shown in Figure 4B, the green fluorescence in the TPs + NaF group was significantly reduced when compared with that in the NaF group (*p* < 0.001) and almost returned to the level in the control group, from which it was inferred that TPs have a significant effect in alleviating the proportion of apoptosis induced by F exposure.

In addition, we also found that TP supplementation decreased the IPEC-J2 cell damage rate induced by NaF via inhibiting LDH’s release from the cytoplasm to the medium (*p* < 0.05) (Figure 4C). As for the level of TJ proteins in the IPEC-J2 cells, TP supplementation improved (*p* < 0.05) the relative mRNA levels of ZO-1 and Occludin when compared with those in the NaF group but significantly inhibited the Claudin-1 level (*p* < 0.001) (Figure 4D). Combined with the above results in Figure 4A–D, it was supposed that TPs contributed to dealing with the cell injury induced by excessive F exposure.

## 4. Discussion

In the absence of effective medication, tissue damage from fluorosis has become a key public health issue. It is well known that long-term high F intake increases oxidative stress levels, inflammatory-related factor secretion, apoptosis, and organelle damage [25,26]. In this study, we found significant dose–response changes in IPEC-J2 cell viability due to the content of F added, which demonstrated that the toxic effects of F increased with the exposure concentration. Many studies have shown that tea or its functional components could inhibit cell proliferation of cancer cells via regulating some cell survival pathways [27,28,29], protecting neurocytes or hepatocytes against oxidative stress-mediated cell death [30,31]. The present study found that the viability of F-exposed cells was increased by 200 mg/L of TP supplementation, which further demonstrated that TPs could promote cell proliferation against apoptosis induced by heavy metals.

The major antioxidant enzymes in the intestine, such as SOD, CAT, and GSH-Px, could protect cell from oxidative stress induced by superoxides [32]. Actually, F could inhibit the activity of antioxidant-related enzymes and decrease some antioxidant contents, all of which lead to excessive oxygen free radical generation and greater oxidative stress [33]. Likewise, F induction not only inhibited the activities of T-SOD, CAT, and GSH-Px but also their transcription levels, as presented in this study. ROS mainly occur due to enzymatic reactions or the byproducts of cellular respiration. Accordingly, antioxidant defenses decline when the redox state is altered by ROS accumulation [18]. To maintain the ROS at an appropriate level, tissues usually use antioxidant components to reduce free radical cytotoxicity and provide major antioxidant defenses against the ROS threat, especially SOD and CAT [34]. The antioxidant activity of TPs partially depends on their activities as ROS scavengers [35]. In this study, it was further verified that TPs are efficient at resisting intestinal oxidative stress induced by F exposure, according to the levels of ROS and the antioxidant-related enzymes of IPEC-J2 cells.

Abnormal oxidation can interfere with reversible oxidation-reduction reactions, which physiologically function in cell proliferation and apoptosis [36]. Mitochondria participate in oxidative-mediated apoptosis [37]. Loss of some antioxidant-related substances from mitochondria would increase ROS generation and lead to colonic epithelial cell apoptosis [38]. It has been reported that ROS generation would lead to mitochondria-mediated apoptosis [39]. Consequently, the available evidence in this study shows that TPs relieved IPEC-J2 cell apoptosis caused by F induction, referring to the results for the TUNEL and MMP, which may have directly reduced LDH’s release from the cytoplasm to the medium, thus attenuating the cell damage rate. Furthermore, it has been proven that F disrupted the intestinal epithelial tight junction integrity [40,41] and TPs help improve intestinal barrier function [42], which confirms that TP supplements may contribute to restoring an intestinal barrier disrupted by F exposure through increasing TJ protein expression in IPEC-J2 cells. However, more research is still needed to determine exactly how TPs lead to susceptibility in IPEC-J2 cells exposed to excessive F.

## 5. Conclusions

Collectively, our results highlight the important role of TPs in protecting intestinal epithelial cells from F-induced oxidative stress via improving the activity of antioxidant enzymes and limiting ROS generation to modify oxidative balance and cell apoptosis. These findings would provide more evidence supporting that TPs could be used as a valuable candidate for preventing F-induced gastrointestinal injury in F-rich areas.

## Figures and Tables

**Table 1:** The primer sequences for RT-qPCR.

Gene	ID	Primer Sequence (5′-3′)	Length (bp)
GAPDH	NM_007393.5	F: TGTCCACCTTCCAGCAGATGT	101
		R: AGCTCAGTAACAGTCCGCCTAGA	
GPX1	NM_214201.1	F: TGGGGAGATCCTGAATTG	184
		R: GATAAACTTGGGGTCGGT	
GPX2	NM_001115136.1	F: TGCAACCAATTTGGACATCAG	122
		R: TTCACGTCACACTTCTGGATAAGG	
GPX3	NM_001115155.1	F: AAACAGGAACCGGGAGACAA	156
		R: AGGACAGGCGTTCTTCAGGAA	
GPX4	NM_214407.1	F: GATTCTGGCCTTCCCTTGC	173
		R: TCCCCTTGGGCTGGACTTT	
CAT	XM_021081498.1	F: CGAAGGCGAAGGTGTTTG	370
		R: AGTGTGCGATCCATATCC	
SOD	XM_021100523.1	F: ATGCTGACGCTGCTCTGTGCTTA	143
		R: TCCTGCCAGATCTCCGTCACTTT	
Occludin	NM_001360536.1	F: CCCCTCTTTCCTTAGGCGAC	168
		R: TTCAAAAGGCCTCACGGACA	
Claudin-1	NM_008770.3	F: TGGTGGACATCCTCATCCTT	190
		R: GCCAGCAGAATAAGGAGCAC	
ZO-1	NM_001417374.1	F: GAGCAGGCTTTGGAGGAGAC	162
		R: TGGGACAAAAGTCCGGGAAG	

## Data Availability

The data presented in this study are available upon request from the corresponding author.
